# Fine Mapping Identifies *SmFAS* Encoding an Anthocyanidin Synthase as a Putative Candidate Gene for Flower Purple Color in *Solanum melongena* L.

**DOI:** 10.3390/ijms19030789

**Published:** 2018-03-09

**Authors:** Mengqiang Chen, Mengyun Xu, Yao Xiao, Dandan Cui, Yongqiang Qin, Jiaqi Wu, Wenyi Wang, Guoping Wang

**Affiliations:** 1 Key Laboratory of Biology and Germplasm Enhancement of Horticultural Crops in South China, Ministry of Agriculture, College of Horticulture, South China Agricultural University, Guangzhou 510642, China; chenmq068@163.com; 2 College of Horticulture, South China Agricultural University, Guangzhou 510642, China; 11116047@zju.edu.cn (M.X.); xiaoyao1990@cau.edu.cn (Y.X.); cuidan0627@163.com (D.C.); yqg_qin@163.com (Y.Q.); wujiaqi_3764@163.com (J.W.); 3 Department of Plant Science, Weizmann Institute of Science, Rehovot 76100, Israel

**Keywords:** anthocyanidin synthase, eggplant, fine mapping, flower color

## Abstract

Anthocyanins are the main pigments in flowers and fruits. These pigments are responsible for the red, red-purple, violet, and purple color in plants, and act as insect and animal attractants. In this study, phenotypic analysis of the purple flower color in eggplant indicated that the flower color is controlled by a single dominant gene, *FAS*. Using an F_2_ mapping population derived from a cross between purple-flowered ‘Blacknite’ and white-flowered ‘Small Round’, *Flower* *Anthocyanidin Synthase* (*FAS*) was fine mapped to an approximately 165.6-kb region between InDel marker Indel8-11 and Cleaved Amplified Polymorphic Sequences (CAPS) marker Efc8-32 on Chromosome 8. On the basis of bioinformatic analysis, 29 genes were subsequently located in the *FAS* target region, among which were two potential *Anthocyanidin Synthase* (*ANS*) gene candidates. Allelic sequence comparison results showed that one *ANS* gene (*Sme2.5_01638.1_g00003.1*) was conserved in promoter and coding sequences without any nucleotide change between parents, whereas four single-nucleotide polymorphisms were detected in another *ANS* gene (*Sme2.5_01638.1_g00005.1*). Crucially, a single base pair deletion at site 438 resulted in premature termination of *FAS*, leading to the loss of anthocyanin accumulation. In addition, *FAS* displayed strong expression in purple flowers compared with white flowers and other tissues. Collectively, our results indicate that *Sme2.5_01638.1_g00005.1* is a good candidate gene for *FAS*, which controls anthocyanidin synthase in eggplant flowers. The present study provides information for further potential facilitate genetic engineering for improvement of anthocyanin levels in plants.

## 1. Introduction

Three major groups of chemical pigments—betalains, carotenoids, and anthocyanins—are responsible for the colors of flowers [1,2,3]. Of these, anthocyanins belong to a large group of phenolic secondary metabolites known as flavonoids, which are responsible for the red, red-purple, violet, purple, and blue colors found in many flowers and fruits [3,4]. Anthocyanins are generally synthesized in the cytosol of epidermal cells via the phenylpropanoid pathway and are subsequently transported to the vacuole or cell walls where they are stored. The biosynthesis of anthocyanins has been well studied in many plant species, and the key regulatory genes and relevant transcriptional factors controlling the pathway have been isolated over the past few decades [1,5,6].

Eggplant (*Solanum melongena* L.), an important horticultural crop worldwide, is currently widely cultivated in Asia, Africa, North America, and Europe. Eggplant not only provides numerous compounds beneficial to health, including vitamins and minerals, but also contains important phytonutrients such caffeic and chlorogenic acids and flavonoids [7,8,9]. Eggplants with purple flowers and fruits are rich in anthocyanins, which are among the most important secondary metabolites and beneficial to human health. Moreover, the color of eggplant flowers is generally linked to yield, fruit coloration, and disease resistance. Therefore, exploring DNA makers or genetic loci related to flower color is considered as an important approach in rapid breeding via marker-assisted selection (MAS).

**Figure 1:**
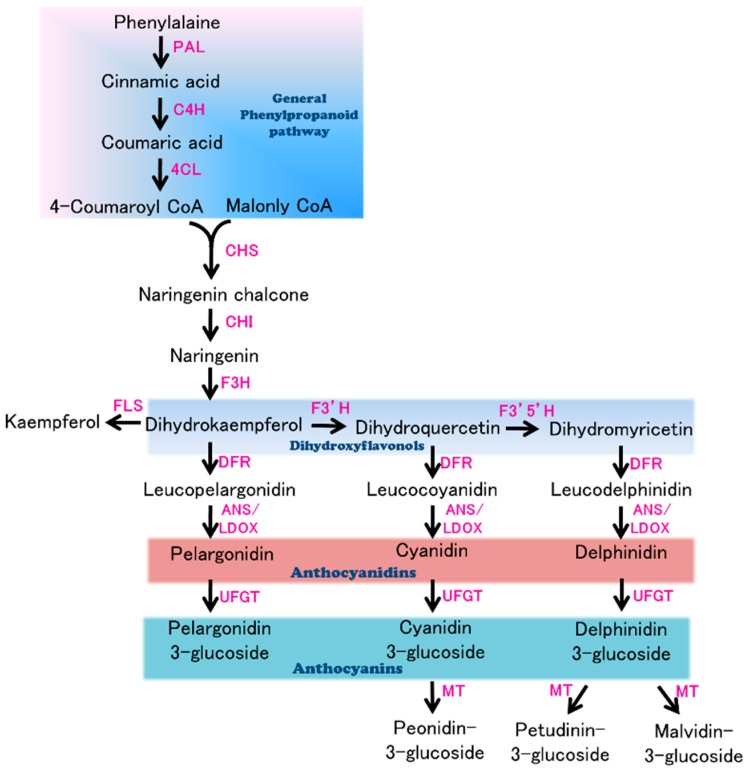
Biosynthesis of anthocyanin in plants. Enzymes are indicated in pink text. Abbreviations: PAL, phenylalanine ammonialyase; C4H, cinnamate-4-hydroxylase; 4CL, 4 coumarate CoA ligase; CHS, chalcone synthase; CHI, chalcone isomerase; F3H, flavanone 3-hydroxylase; FLS, flavonol synthase; F3′H, flavanone 3′-hydroxylase; F3′5′H, flavanone 3′, 5′-hydroxylase; DFR, dihydroflavonol 4-reductase; ANS/LDOX, anthocyanidin synthase/leucoanthocyanidin dioxygenase; UFGT, UDP-flavonoid glucosyl transferase; MT, methyl transferase.

In recent years, with the release of the draft genomes of many plants, several genes and quantitative trait loci (QTL) that control flower and fruit color have been reported in different plant species [10,11,12,13,14]. Furthermore, given that anthocyanin biosynthesis is currently an important focus of plant secondary metabolism, intensive efforts have been made to identify the key genes of the respective biosynthetic pathways. The anthocyanin biosynthetic pathway (Figure 1), which starts with phenylalanine, contains five key enzyme genes: *chalcone synthase* (*CHS*), *chalcone isomerase* (*CHI*), *flavanone*-*3*-*hydroxylase* (*F3H*) or *flavanone 3′*-*hydroxylase* (*F3′H*) *or flavanone 3′*, *5′*-*hydroxylase* (*F3′5′H*), *dihydroflavonol 4*-*reductase* (*DFR*), and *anthocyanidin synthase* (*ANS*)*.* Notably, ANS, a 2-oxoglutarate and Fe^2+^-dependent oxygenase, which is the last key enzyme in the anthocyanin biosynthetic pathway, catalyzes the transformation of colorless leucoanthocyanidin to anthocyanidins and proanthocyanidins. Functional defects in (or the absence of) *ANS* affects plant color formation, resulting in colorless or white organs, suggesting its importance in the formation of color in plants [15]. A notable physiological function of the anthocyanin pigments is recruitment of polleninator and seed dispersers [5], moreover, anthocyanin pigments play an important role in signaling between plants and micobes, UV protection [5]. Thus, it is desirable to explore anthocyanin biosynthesis mechanism in plants. In the present study, a *Flower Anthocyanidin Synthase* (*FAS*) locus was cloned from a 165.6-kb region on Chromosome 8 using an F_2_ mapping population derived from a cross between the cultivars Blacknite (BN: purple flower, purple fruit) and Small Round (SR: white flower, green fruit). On the basis of bioinformatic and expression analyses, a mutation in an *ANS* gene, resulting in premature termination of the predicted polypeptide, was shown to be responsible for the white color in eggplant flowers.

## 2. Materials and Methods

### 2.1. Plant Materials and Mapping Populations

F_2_ mapping population, containing 3133 lines, was developed from a cross between the cultivars Blacknite (BN: purple flower, deep purple fruit, purple stem and purple calyx, collected in Australia) and Small Round (SR: white flower, green fruit, green stem and green calyx, originated in south of china), and used for linkage and genetic mapping analysis. An additional 14 purple or white flower cultivars were used for mutation analysis in the target region. All eggplants were grown on the experimental field of South China Agricultural University in Guangzhou (23°20′ N 113°30′ E, subtropical climate, China) from spring to summer.

### 2.2. DNA Extraction and SSR Identification

Genomic DNA was extracted from young leaves using the Cetyl trimethylammonium bromide (CTAB) method with some modifications. All of the primers used in this study were designed by SSRHunter1.3 (http://en.bio-soft.net/dna/SSRHunter.html) and Primer Premier 5.0 software (Premier Biosoft International, Palo Alto, CA, USA), then synthesized by Sangon Bitotech Co., Ltd. (Guangzhou, China). All primers used in this study are listed in Supplemental Table S1. Each 10 μL PCR reaction contained 5.6 μL water, 1.0 μL 10× buffer (Mg^2+^), 1 μL dNTPs (10 mM), 1 μL each of upstream and downstream primers (1 μM), 0.4 μL *Taq* DNA polymerase (10 U/μL). The PCR amplification was performed with the following program: 94 °C for 5 min followed by 36 cycles of 94 °C for 30 s, 55 °C for 30 s, and 72 °C for 1 min with a final extension at 72 °C for 10 min. Subsequently, the PCR amplification products were subjected to electrophoresis in a 6% polyacrylamide gel.

### 2.3. Mapping Strategy and Linkage Analysis

Over 1500 eggplant simple sequence repeat (SSR) markers developed by our laboratory were used to screen for polymorphism between parental lines. Then the polymorphic markers were used to genotype the individuals of F_2_ mapping population. A group of 40 individual plants were randomly selected for preliminary mapping. The chromosome position of the linked marker EMJ316 was inferred by the similarity of its marker sequence with tomato genome sequence and the genome between tomato and eggplant. Eggplant draft genome sequence was used to develop a series of new SSR markers around the target region. The obtained polymorphic SSR markers were subsequently used to genotype 174 F_2_ plants. Preliminary mapping analysis was performed with Jounmap3.0 software (Plant Research International BV, Wageningen, The Netherlands).

When the available SSR markers in the target region were exhausted, BN and SR were submitted to re-sequence on the Illumina DNA sequencing platform by Biomaker Technology Company (Beijing, China). According to the information of sequence polymorphism within the *FAS* target region, InDel and Single nucleotide polymorphism (SNP)-based derived cleaved amplified polymorphic sequence (dCAPS) markers were continuously designed. Two robust boundary markers spanning the *FAS* region were used to screen 3133 F_2_ population for recombination plants to narrow the interval containing target gene. The genetic distance was calculated by the Kosambi function.

### 2.4. Candidate Gene Prediction and Identification

Candidate gene prediction was performed by the online program FGENESSG, eggplant and tomato genome browser (http://eggplant.kazusa.or.jp/, https://solgenomics.net/organism/Solanum_lycopersicum/genome), and BLASTx of NCBI (National Center for Biotechnology Information, https://blast.ncbi.nlm.nih.gov/Blast.cgi). To clone sequences of the two potential candidate genes, primers were designed by software Primer Premier 5.0 in the target region. Gene alignments among different materials were performed using CLUSTALX1.8 (CNRS/INSERM/ULP, Illkirch Cedex, France) with default settings. All eggplant amino acid sequences were obtained from online draft genome sequence database (http://eggplant.kazusa.or.jp/).

### 2.5. Quantitative Real-Time RT-PCR Analysis of Candidate Genes

Total RNAs were extracted from different tissues (root, stem, leaf, flower, and fruit) of BN, SR and its crossbred F_1_ plants using Hipure Plant RNA kits according to the manufacturer’s instructions. Primers were designed by software Primer Premier 5.0. Quantitative real-time RT-PCR (qRT-PCR) analysis was conducted with the Lightcycler 480 machine (Roche, Sussex, UK) using SYBR Green I. The expression of genes was calculated with 2^−ΔΔ*C*t^ method. The eggplant *Glyceraldehyde*-*3*-*phosphate dehydrogenase* (*GADPH*) gene was used as an internal reference. Each experiment was had three biological and technical repetitions. The specific primers for qRT-PCR are listed in Supplementary Table S1.

## 3. Results

### 3.1. Inheritance of SmFAS

**Figure 2:**
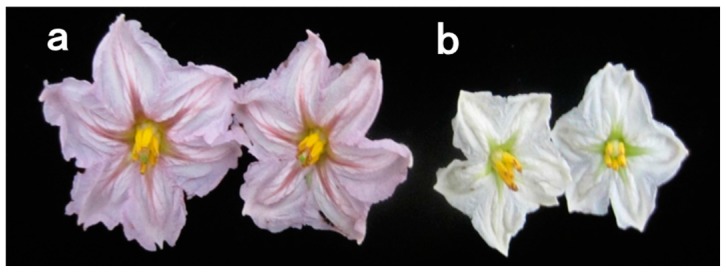
Flower color of parental lines. (**a**) The purple flower in Blacknite (BN); (**b**) the white flower in Small Round (SR).

Eggplant cultivars Blacknite (BN) and Small Round (SR) were used to generate F_1_ and F_2_ population (Figure 2). All the F_1_ plants of reciprocal crosses exhibited purple-colored flowers, suggesting the dominant nature of purple to while flower color. Of 174 F_2_ individuals, flower color segregated as 137 purple to 37 white, which is consistent with a 3:1 Mendelian segregation ratio (Table 1), indicating that the flower color in eggplant is controlled by a single dominant gene, which we designated as *SmFAS* (*Flower Anthocyanidin Synthase*).

### 3.2. Linkage Mapping of SmFAS

**Figure 3:**
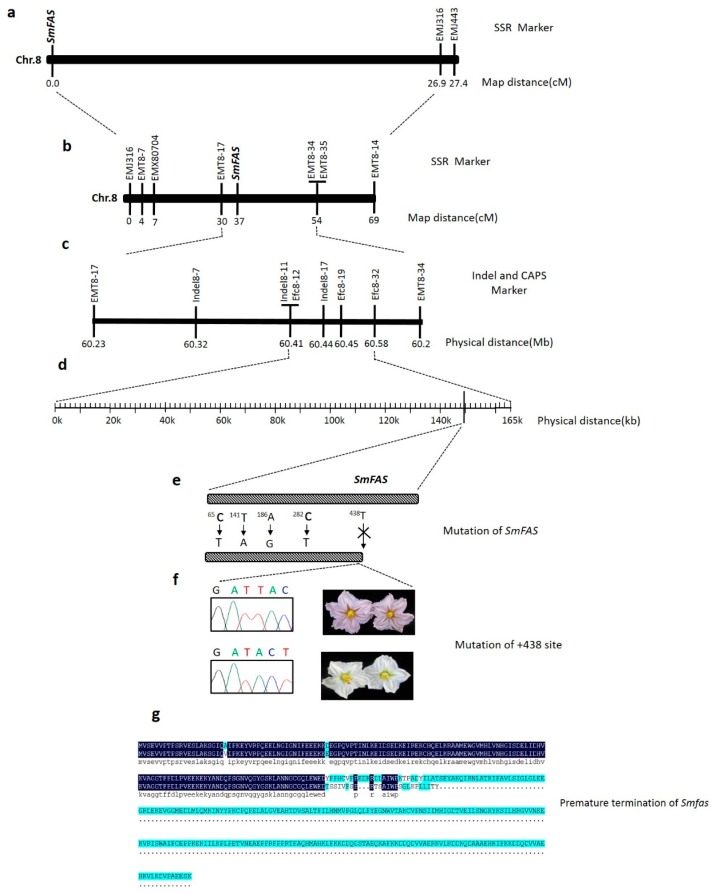
Genetic linkage map of *Flower Anthocyanidin Synthase (FAS)* locus. (**a**) Chromosomal location of *FAS* locus; (**b**,**c**) framework map based on F_2_ family; (**d**) high-resolution map of *FAS* locus into a ~165.6 kb between Indel8-11 and Efc8-32; (**e**) the structure of *FAS* shows that four single nucleotide polymorphisms (SNPs)were detected between parents in the coding sequence (CDS), and a single base pair deletion in site 438; (**f**) the site 438 of *FAS* in parents and their sequencing chromatograms—upper panel: BN; lower panel: SR. (**g**) the mutation in site 438 resulted in premature termination of *FAS*.

Initially, 1052 eggplant SSR markers developed in our laboratory were used to screen polymorphic markers. The polyacrylamide gel electrophoresis (PAGE) results revealed that 761 (72.9%) markers were non-polymorphic, 211 (20.0%) could not be amplified, and the remaining 74 (7.7%) appeared to be polymorphic between parental lines. We used these 74 polymorphic SSR makers to genotype 40 random F_2_ mapping individuals. Linkage analysis indicated that the *SmFAS* locus was linked to the EMJ316 marker, with a genetic distance is 26.9 cM (Figure 3a). Detailed information of the map locations of eggplant SSR polymorphic markers is presented in Figure S1.

To identify markers flanking *SmFAS*, a series of new SSR markers in the target region were continuously developed near the EMJ316 marker. Seven new polymorphic SSR markers were subsequently used to screen 174 F_2_ plants. Among these, ETM8-17 and ETM8-34 were located 4.0.0 and 17.0 cM from the *SmFAS* locus (Figure 3b). Through a comparison with the eggplant draft genome, ETM8-17 and ETM8-34 were consistent with the scaffolds of Sme2.5_03884.1 and Sme2.5_05139.1, and the physical distance between the two markers was ~423.6 kb (Figure 3c). 

To precisely narrow down the region surrounding *SmFAS*, Indel marker Indel8-7 and SSR marker ETM8-35 were used to genotype 3133 F_2_ plants, and 69 recombinant plants were identified. Finally, three Indel markers and three CAPS makers between ETM8-17 and ETM8-34 were developed to identify the 69 recombinant plants. The results showed that Indel8-11, Efc8-19, Efc8-12, Indel8-17, and Efc8-32 were in complete genetic linkage with the *SmFAS* locus without any recombination events. Thus, the *SmFAS* locus was located in a region of ~165.6 kb between Indel8-11 and Efc8-32 (Figure 3d).

### 3.3. Candidate Genes for Purple Flower Color in Eggplant

On tomato genome, there were 24 annotated genes within the corresponding region spanned by marker Indel8-11 and Efc8-32, and one gene *Solyc08g080040.2.1* was predicted as anthocyanidin synthase. Six eggplant scaffolds located in the target region contained 29 genes. Interestingly, two *ANS* genes (*Sme2.5_01638.1_g00003.1* and *Sme2.5_01638.1_g00005.1*) were predicted. 

Among the 29 predicted genes of eggplant, the coding sequence (CDS) of 22 genes including *Sme2.5_01638.1_g00003.1* had no any nucleotide difference between two parents according to the re-sequencing information (Table 2). The CDS of six genes existed single nucleotide polymorphisms (SNPs) which resulted in no or one amino acid substitution but possibly did not cause the loss of gene function. However, four SNPs were detected for the gene *Sme2.5_01638.1_g00005.1*. Among these, mutations at positions +65 bp and +141 bp resulted in changes in the 23rd and 47th amino acid residues, from Ala (A) to (Val) V and from (Asp) D to (Glu) E, respectively (Figure 3e,f). More crucially, one single base pair deletion at site 438 resulted in premature termination of *SmFAS* (Figure 3g), which leads to a malfunction of *ANS*. These preliminary results indicate that *Sme2.5_01638.1_g00005.1* is the best candidate gene for *SmFAS*.

**Figure 4:**
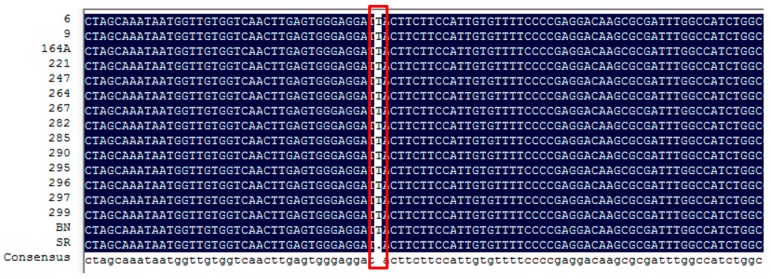
Comparison of *Sme2.5_01638.1_g00005.1* (*ANS*) gene in 16 eggplants with purple and while flower.

To explore whether the color of flower is consistent with the nucleotide insertion/deletion mutation in *Sme2.5_01638.1_g00005.1*, we further PCR-amplified the full length of *FAS* gene of 16 eggplant cultigens with purple or white flowers (Table 3). The SNPs and insertion/deletion between two parents were confirmed once again. For all purple flower eggplants, none SNP was found in *Sme2.5_01638.1_g00005.1* compared with BN; whereas in two other white flower cultivars, the deletion in 438 site resulted in premature termination of *FAS* was undetectable (Figure 4), suggesting that the white flower caused by the deletion of site 438 of *SmFAS* was genotype-specific in cultivar SR.

### 3.4. Expression Analysis of Two ANS Genes in Various Materials and Tissues

**Figure 5:**
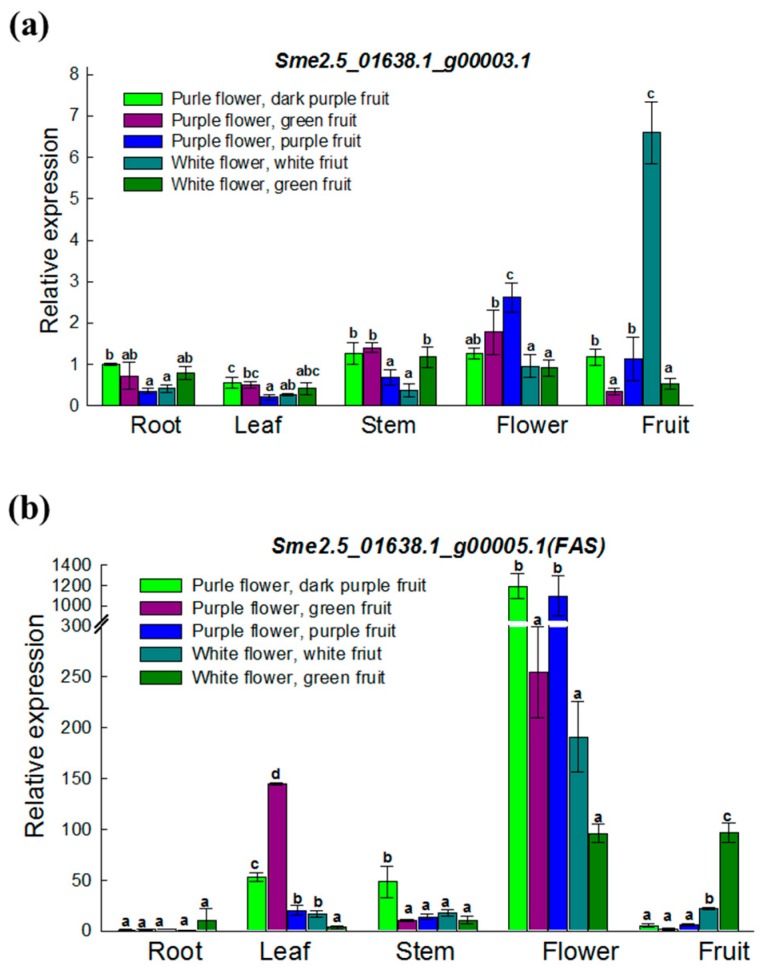
Expression analysis of the two *ANS* candidates in parental lines, F_2_ progenies with different phenotypes in various tissues (root, leaf, stem, flower, fruit) by qRT-PCR. (**a**) *Sme2.5_01638.1_g00003.1*; (**b**) *Sme2.5_01638.1_g00005.1*. Data are expressed by mean of three biological replicates with error bars indicating the SD. Letter represent significant difference among the five phenotypes using (Analysis of variance) ANOVA. a, b, c and d: *p* < 0.05, Student Newman-Keuls test.

As described in the previous paragraph, there are two potential candidate *Anthocyanidin Synthase* (*ANS*) genes in the region targeted in this study. We used qRT-PCR to further investigate the expression levels of both candidate *ANS* genes in different tissues (roots, stems, leaves, flowers, and fruits) from parental lines and their F_2_ progenies showing various phenotypes, such as white flower with green fruit, purple flower with green fruit, purple flower with purple-black fruit. The results revealed *Sme2.5_01638.1_g00003.1* to be expressed very weakly in all organs without significant difference (Figure 5a). Whereas *Sme2.5_01638.1_g00005.1* displayed strong expression, particularly in flowers compared with roots, stems, leaves, and fruit in all materials (Figure 5b). Notably, *Sme2.5_01638.1_g00005.1* showed significant differences in expression profiles between purple and white flowers, which was consistent with the flower phenotype. This further indicates that *Sme2.5_01638.1_g00005.1* is the most probable candidate gene for *FAS*. Moreover, the data also indicated that different types of ANS may be present in flowers and fruits, since expression of *SmFAS* is lower in green fruit compared with purple fruit. 

## 4. Discussion

Flower color, which is used to attract insect pollinators, is an important trait of plants. Anthocyanidins are the most abundant flavonoid pigments that determine the flower color. The mechanisms behind the accumulation of floral pigments have been well-studied in several plants [1,5,16,17]. In the present study, using map-based cloning strategy, we delimited the *Flower Anthocyanidin Synthase* (*FAS*) locus to a 165.6-kb region on chromosome 8 in *Solanum melongena* L. Sequencing and expression analysis revealed that *FAS* encoded an ANS. Results from this study support the notion that the dominant gene, *FAS*, controls the flower color in *Solanum melongena* L., which is consistent with the results of previous studies [9,12,13].

Recently, a locus controlling the purple corolla in eggplant was mapped to chromosome 12 between two markers, gg9149_779 and emxC0904 [9]; this is different from the locus identified by us, which is located on chromosome 8. In the study by Hirakawa, the candidate gene responsible for the purple corolla was a MYB-like transcription factor, which acts as an activator or repressor of gene expression, controlling the color of flowers in eggplant. In contrast, the gene controlling the purple flower trait identified by us is *ANS*, which is different from the transcriptional factor controlling the flower color in eggplant. In flowers, three kinds of transcriptional factors (R2R3-MYB, bHLH, and WD40) and their combinations that control anthocyanin synthesis have been intensively studied [18,19,20,21]. Among these, R2R3-MYB is generally considered to be closely associated with the biosynthesis and regulation of anthocyanins. For example, in gerbera plants, the overexpression of *MYB10* led to a notable increase in the accumulation of anthocyanin [22,23]. 

Barchi et al. (2012) used an F_2_ population to map the quantitative trait loci (QTLs) for seven traits associated with anthocyanin content and identified 11 different QTL regions [24]. Among these, two loci located in chromosome 8, namely adaxial/abaxial leaf lamina anthocyanin and calyx anthocyanin. Subsequently, Cericola et al. (2014) identified 12 loci on 9 chromosomes responsible for anthocyanin pigmentation and fruit color by performing a genome-wide association analysis using 191 eggplant accessions and 384 SNP loci markers [25]. Notably, loci identified by Barchi and Cericola group were far away from the *FAS* mapped in this study. The reason for this is that the flower color of two parents in Barchi’s study was purple of different levels. Whereas in Cericola’s study, some eggplant with white flower were used in the study, locus of *FAS* has not yet to be found, the possible reason could be attributed to the low frequency of white flower caused by function loss of *FAS* in natural population. In this study, two of three cultigens with white flower without deletion at position 438 of *FAS* gene. Similarly, the white flower loci mapped on chromosome 12 by Hirakawa et al. (2014) were also not detected in Cericola’s study [9]. Collectively, the molecular mechanism underlying for white flower in eggplant might be complex.

**Figure 6:**
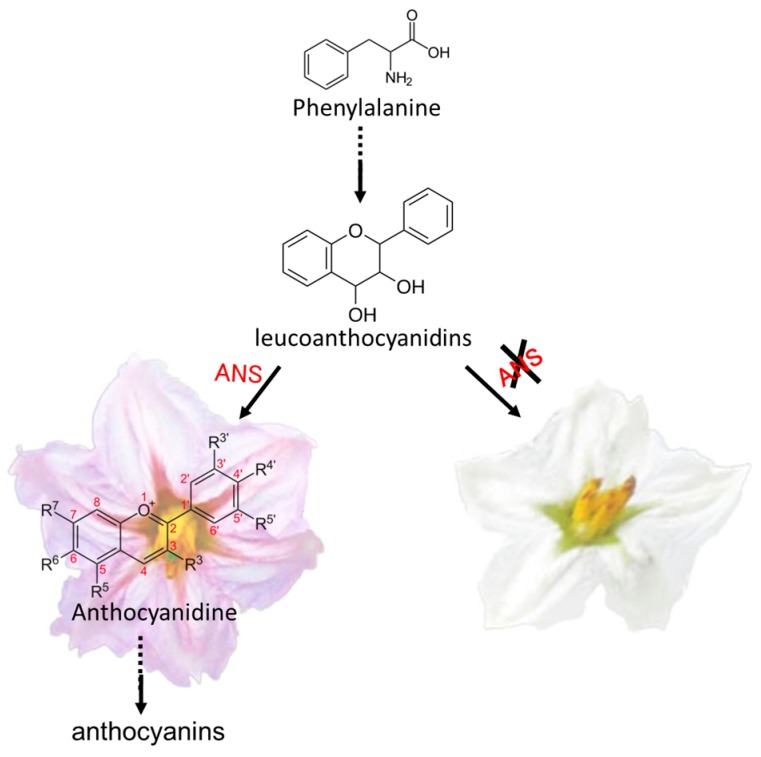
Functional defects or mutations in *ANS* influence the flower color formation, leading to white flower. The dashed arrow indicates several enzymatic reactions. The arrow represents specific enzymatic reaction. ”**X**” represents fail to synthesize anthocyanidin due to malfunction of *ANS* gene.

Generally, the visible accumulation of anthocyanidins reflects the activity of the enzymes involved in the biosynthetic pathway [26]. We detected the expression of *FAS* in the parental lines and in their F_2_ progenies with different phenotypes, such as white flower with green fruit, purple flower with green fruit, and purple flower with purple-black fruit. Furthermore, we also checked the expression in different tissues (roots, stems, leaves, flowers, and fruits), the results showed that *FAS* was strongly expressed in purple flowers (Figure 4b). In this respect, our results are consistent with those reported in the flowers of different species, such as maize, snapdragon, and petunia [27,28,29]. Generally, the expression of *ANS* was in consonance with the color shades in fruits and flowers. For example, in the purple organs, the expression was specifically upregulated compared to that in the pink or white organs in different plant species [26]. Similar results were also obtained in eggplant in our study. Moreover, during fruit maturation, the expression levels of anthocyanidin pathway genes, including *ANS*, were increased and were related to the accumulation of anthocyanidins in many plants, such as bilberry, mock strawberry, pea, snapdragon, and petunia [27,30,31,32]. These results were consistent with those observed for our candidate gene, *SmFAS*. Overall, these findings suggest that *FAS* is a gene associated with late stage of anthocyanin biosynthesis pathway, and is involved in the regulation of the flower color in eggplant (Figure 6).

Given the importance of ANS in the anthocyanidin biosynthesis pathway, the role of *ANS* has been intensively studied in several plants, such as *Arabidopsis*, apple, *Lisianthus*, strawberry, and pomegranate [1,33,34]. Functional defects or mutations in ANS often influence the color formation, leading to colorless or white organs. In apple, silencing of *ANS* led to significant loss of anthocyanins in transgenic plants. In *Lisianthus*, a 94 bp deletion mutation and frame shift in the *ANS* gene was confirmed to be associated with acyanic flowers [35]. Notably, Ben-Simhon et al. (2015) recently demonstrated that an insertion in the coding region of *PgLDOX* (leucoanthocyanidin dioxygenase, also known as *ANS*) resulted in white anthocyanin-less phenotype in pomegranate [36]. This trait was further proven to be controlled by a recessive signal gene. In the present study, we observed that another single base pair deletion at position 438 of *ANS* resulted in its premature termination, and the expression levels of *ANS* in F_2_ progenies correlated with different phenotypes, suggesting that this mutation disrupts the normal transcription of gene, leading to the failure of anthocyanidin synthesis and generation of white flowers. We further checked the mutation site in 14 eggplant cultivars with purple and white flowers, and noticed that the mutation at position 438, responsible for the premature termination of anthocyanidin synthase, was undetectable in all these eggplant cultivars, suggesting that mutation in *ANS* results in the white flower phenotype in the eggplant cultivar, SR. Moreover, the purple flower trait was identified to be controlled by a dominant signal gene. Taken together, our findings provide a better understanding of anthocyanin biosynthesis in eggplant and may facilitate genetic engineering of plans for enhanced anthocyanin content. 

## Figures and Tables

**Table 1:** Segregation of flower color in the F_1_ and F_2_ population

Population	Plant Tested	Purple:White	Mendelian Expectations	X^2^	*p*
BN (purple) × SR (white)					
F_1_	all	all purple	1:0		
F_2_	174	137:37	3:1	1.295	0.255

BN: Blacknite; SR: Small Round.

**Table 2:** Candidates’ annotations in the *Flower Anthocyanidin Synthase (FAS* )target region on Chromosome 8

Scaffold	CDS	Gene	Arabodopsis Homologs	Annoations	Polymorphism
Sme2.5_01332.1	Sme2.5_01332.1_g00003.1	*Solyc08g079820.2.1*	AT4G11980.1	nudix hydrolase 14	None
	Sme2.5_01332.1_g00004.1	*Solyc08g079790.1.1*	AT1G12500.1	sugar phosphate/phosphate translocator	None
	Sme2.5_01332.1_g00005.1	*Solyc08g079780.1.1*	AT3G17675.1	mavicyanin-like protein	1 SNP
	Sme2.5_01332.1_g00006.1	*Solyc08g079770.2.1*	AT1G12480.1	guard cell S-type anion channel	None
	Sme2.5_01332.1_g00007.1	*Solyc08g079760.2.1*	AT4G22860.1	protein TPX2-like	None
	Sme2.5_01332.1_g00008.1	*Solyc08g079750.2.1*	AT1G62960.1	aminotransferase ACS10	None
	Sme2.5_01332.1_g00009.1	*Solyc08g079740.2.1*	AT1G12460.1	LRR receptor-like serine/threonine-protein kinase	None
	Sme2.5_01332.1_g00010.1	*Solyc12g044400.1.1*	AT3G16290.1	inactive ATP-dependent zinc metalloprotease FTSH 12, chloroplastic	None
	Sme2.5_01332.1_g00011.1	*Solyc08g079730.1.1*	AT4G22790.1	protein DETOXIFICATION 56-like	None
Sme2.5_20506.1	Sme2.5_20506.1_g00001.1	*Solyc08g079790.1.1*	AT1G12500.1	sugar phosphate/phosphate translocator	None
	Sme2.5_20506.1_g00002.1				None
	Sme2.5_20506.1_g00003.1				None
Sme2.5_25093.1	Sme2.5_25093.1_g00001.1	*Solyc08g079820.2.1*	AT4G11980.1	nudix hydrolase 14	None
	Sme2.5_25093.1_g00002.1	*Solyc08g079830.2.1*	AT1G12520.1	copper chaperone for superoxide dismutase	None
Sme2.5_14718.1	Sme2.5_14718.1_g00001.1	*Solyc08g079850.1.1*	AT1G04110.1	subtilisin-like protease SBT1.2	2 SNPs
Sme2.5_05247.1	Sme2.5_05247.1_g00002.1	*Solyc08g080010.1.1*	AT1G04110.1	subtilisin-like protease SBT1.7	None
	Sme2.5_05247.1_g00003.1	*Solyc08g080000.2.1*		uncharacterized	None
	Sme2.5_05247.1_g00004.1	*Solyc08g079980.1.1*	AT5G67360.1	subtilisin-like protease SBT1.7	None
	Sme2.5_05247.1_g00005.1	*Solyc08g079970.1.1*	AT5G67360.1	subtilisin-like protease SBT1.7	None
	Sme2.5_05247.1_g00006.1	*Solyc07g065420.1.1*	AT5G06990.1	protein MIZU-KUSSEI 1	None
	Sme2.5_05247.1_g00008.1	*Solyc08g079870.1.1*	AT5G67360.1	subtilisin-like protease SBT1.7	None
	Sme2.5_05247.1_g00009.1	*Solyc08g079870.1.1*	AT1G04110.1	subtilisin-like protease SBT1.7	1 SNP
	Sme2.5_05247.1_g000010.1	*Solyc08g079850.1.1*	AT2G05920.1	subtilisin-like protease SBT1.7	3 SNPs
	Sme2.5_05247.1_g000011.1			subtilisin-like protease SBT1.7	2 SNPs
Sme2.5_01638.1	Sme2.5_01638.1_g00002.1	*Solyc08g080030.2.1*	AT1G12550.1	glyoxylate/hydroxypyruvate reductase HPR3-like	None
	Sme2.5_01638.1_g00003.1	*Solyc08g080040.2.1*	AT4G22880.2	*Anthocyanidin Synthase* (*ANS*) gene	None
	Sme2.5_01638.1_g00004.1	*Solyc05g023820.2.1*	AT3G48800.1	tuberosum myb-like protein	2 SNPs
	Sme2.5_01638.1_g00005.1	*Solyc08g080040.2.1*	AT4G22880.2	*Anthocyanidin Synthase* (*ANS*) gene	4 SNPs and 1 InDel
	Sme2.5_01638.1_g00006.1	*Solyc08g080050.2.1*	AT4G22890.2	PGR5-like protein	None

CDS: coding sequence; TPX2: targeting protein for Xklp2 (Xenopus kinesin-like protein 2); LRR: Leucine-rich repeat receptor; FTSH: filamentation temperature sensitive; PGR5: Proton Gradient Regulation5; InDel: insertion-deletion.

**Table 3:** Sample of 16 eggplant cultigens with purple and white flower used for *Flower Anthocyanidin Synthase* (*ANS*) mutation identification.

Eggplant Cultivar	Color of Flower	Color of Peel
6	purple	green
9	purple	white
164A	purple	white
247	purple	white
264	purple	green
267	purple	green
282	purple	green
285	purple	white
290	purple	green
295	purple	white
296	purple	white
297	purple	white
BN	purple	purple
221	white	green
299	white	green
SR	white	green

## Supplementary Materials

Supplementary materials can be found at http://www.mdpi.com/1422-0067/19/3/789/s1.
