# Supplementary material for: Fine Mapping Identifies SmFAS Encoding an Anthocyanidin Synthase as a Putative Candidate Gene for Flower Purple Color in Solanum melongena L
Source: Int J Mol Sci. 2018 Mar 9;19(3):789. doi: 10.3390/ijms19030789 (PMC5877650; doi:10.3390/ijms19030789)
Supplement: Supplementary file 1 [file ijms-19-00789-s001.docx]

**Supplementary Materials: Premature Termination of *Flower Anthocyanidin Synthase* *(FAS)* Leads to the Loss of Anthocyanin Accumulation in Eggplant Flowers**

Mengqiang Chen, Mengyun Xu, Yao Xiao, Dandan Cui, Yongqiang Qin, Jiaqi Wu, Wenyi Wang and Guoping Wang


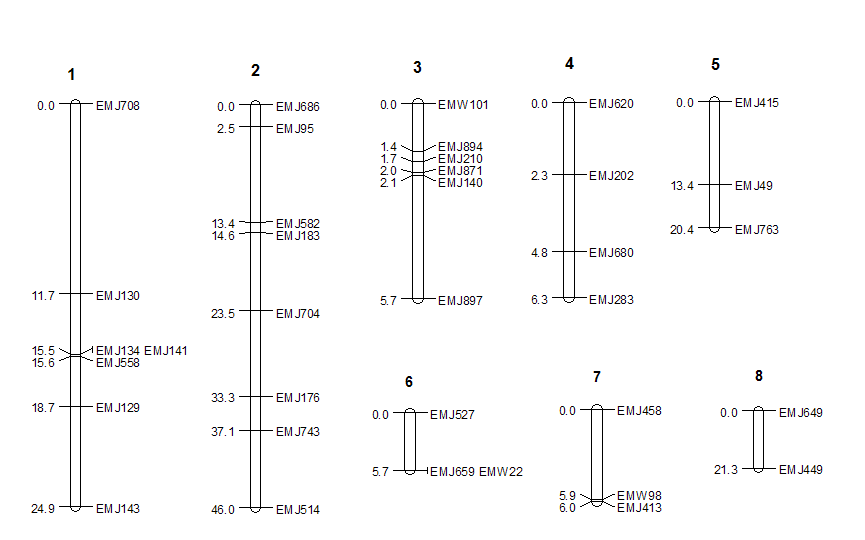


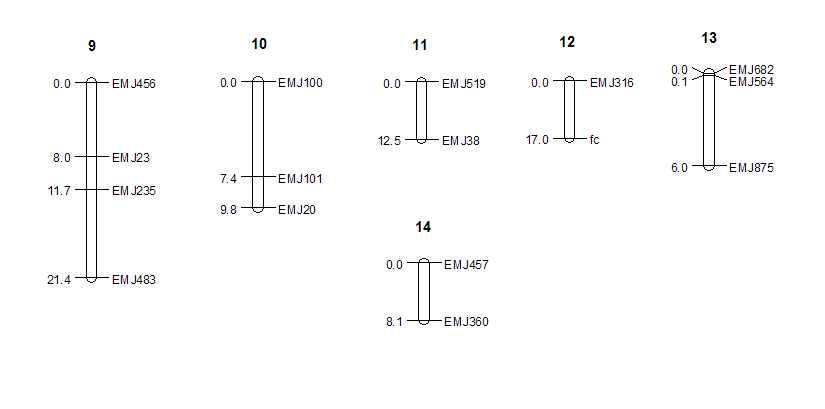


**Figure S1.** The distribution of SSR makers on different chromosome in eggplant.

**Table S1.** Primers used in this study.
